# Associations of the triglyceride-glucose index and its composite indices with mortality in adults with T2DM

**DOI:** 10.3389/fnut.2026.1822295

**Published:** 2026-05-11

**Authors:** Honglin Liu, Yingqi Hou, Shunfu Yu, Zhuchao Wu, Jinjin Liu, Wentao Shi, Guangyou Yan

**Affiliations:** 1 Department of Clinical Laboratory, Zhongshan Second People’s Hospital, Zhongshan, Guangdong, China; 2 Department of Clinical Laboratory, The Third People’s Hospital of Liupanshui, Liupanshui, Guizhou, China; 3 Department of Clinical Laboratory, Changchun University of Chinese Medicine, Changchun, China; 4 Nanjing Medical University, Nanjing, China; 5 Zhabei Central Hospital, Jing’an, Shanghai, China; 6 Clinical Research Unit, Shanghai Ninth People’s Hospital, Shanghai Jiao Tong University School of Medicine, Shanghai, China

**Keywords:** all-cause mortality, T2DM, TyG index, TyG-ABSI, TyG-WWI

## Abstract

**Background:**

The triglyceride-glucose (TyG) index and its composite indices have emerged as potential markers for metabolic risk, but their prognostic value for mortality in middle-aged and older adults with T2DM remains incompletely defined.

**Methods:**

We analyzed data from 1,940 adults with T2DM (aged ≥45 years) who underwent routine health examinations. Baseline characteristics were described using mean (SD), median (IQR), and count (%). Cox proportional hazards regression models were constructed to evaluate associations of TyG-related indices (TyG-WC, TyG-WHR, TyG-WHtR, TyG-ABSI, TyG-WWI) with all-cause mortality, with adjustment for confounders. Restricted cubic splines (RCS) were used to explore nonlinear relationships, and time-dependent ROC curves assessed predictive performance. Subgroup analyses were stratified by age, sex, smoking, drinking, and hypertension status.

**Results:**

During follow-up, 312 (16.1%) participants died. In fully adjusted models, TyG-WC, TyG-WHtR, TyG-ABSI, and TyG-WWI were independently associated with increased mortality (HR range: 1.21–1.44, all *p* < 0.05), with TyG-WHtR showing the strongest association (HR = 1.44, 95%CI: 1.21–1.70). RCS analyses revealed significant J-shaped nonlinear relationships for these composite indices (all *p* for nonlinear <0.01). Time-dependent AUCs indicated moderate predictive performance, with TyG-ABSI and TyG-WWI achieving the highest values. Subgroup analyses confirmed consistent associations in participants aged >65 years, non-drinkers, non-smokers, and those without hypertension.

**Conclusion:**

TyG composite indices, particularly TyG-WHtR, TyG-ABSI, and TyG-WWI, are independent predictors of all-cause mortality in middle-aged and older adults with T2DM, offering potential utility for long-term mortality risk stratification.

## 1 Introduction

Diabetes mellitus represents a leading global contributor to disability and premature mortality, affecting an estimated 589 million adults aged 20–79 years worldwide in 2024, corresponding to 11.11% of this age group (1). The global burden of diabetes is projected to increase by 44.8% from 2024 to 2050, with overall prevalence expected to reach 12.96% and the total number of affected individuals rising to 853 million, emphasizing the critical need for effective preventive and clinical management strategies (1). Mounting evidence has indicated that metabolic disturbances and related diabetic complications substantially elevate the risk of all-cause mortality among affected individuals (2–4). Accordingly, the early identification of patients at high risk of adverse mortality outcomes holds profound clinical and public health significance.

Metabolic disorders, defined by interconnected risk factors including abdominal obesity, elevated blood glucose, high blood pressure, and dyslipidemia (5), are extremely common in individuals diagnosed with type 2 diabetes mellitus (T2DM) (6, 7). Among the various metabolic indicators currently available, the triglyceride-glucose (TyG) index and its associated composite indices—such as the triglyceride-glucose-waist-to-thigh ratio (TyG-WTR), triglyceride-glucose-waist-to-height ratio (TyG-WHtR), and triglyceride-glucose-waist circumference (TyG-WC)—have become increasingly recognized as simple, cost-effective, and dependable markers for insulin resistance and cardiometabolic risk (8, 9). As a valid substitute for insulin resistance, the TyG index can be readily computed using routine clinical parameters, eliminating the need for extra testing expenses (10). Cumulative research has confirmed that the TyG index correlates with a broad spectrum of cardiovascular and metabolic diseases; it not only predicts the risk and severity of these conditions but also their long-term prognosis (11–13). Prior investigations have illustrated that TyG-related indices are linked to mortality in various patient cohorts, including those suffering from chronic kidney disease and non-alcoholic fatty liver disease (14–17). Growing evidence further indicates that insulin resistance, as reflected by the TyG index, may promote systemic inflammation, oxidative stress, and vascular impairment, which in turn deteriorate the prognosis of patients with T2DM (12, 17, 18). Nevertheless, most earlier studies exploring the relationship between TyG-related indices and mortality have concentrated on the general population or patients with other chronic illnesses (e.g., chronic kidney disease, non-alcoholic fatty liver disease), leaving a gap in comprehensive research among adults with T2DM. Furthermore, the comparative assessment of the predictive efficacy of different TyG-based composite indices (e.g., TyG-WTR versus TyG-WHtR) for all-cause mortality in this specific patient group remains incompletely understood.

Considering the high incidence of metabolic dysfunction and the heightened risk of all-cause mortality among adults with T2DM, assessing the associations of TyG-related indices—including TyG-based composite indices combined with anthropometric parameters—with mortality in this population may offer valuable insights for early risk stratification and potential clinical practice. Data for this study were obtained from the Shanghai Center for Disease Control and Prevention, which has developed a comprehensive, standardized diabetes surveillance system covering the entire city. This system systematically documents diabetes-related information, metabolic indicators, and mortality data through community health management, hospital diagnoses, and death registration records. Thus, the objective of this study was to comprehensively explore the associations between TyG-related indices and all-cause mortality among adults with T2DM using this high-quality surveillance data. The results of this research are anticipated to provide practical guidance for clinical risk evaluation, intervention, and management of patients with T2DM.

## 2 Method

### 2.1 Study design and populations

**Figure 1:**
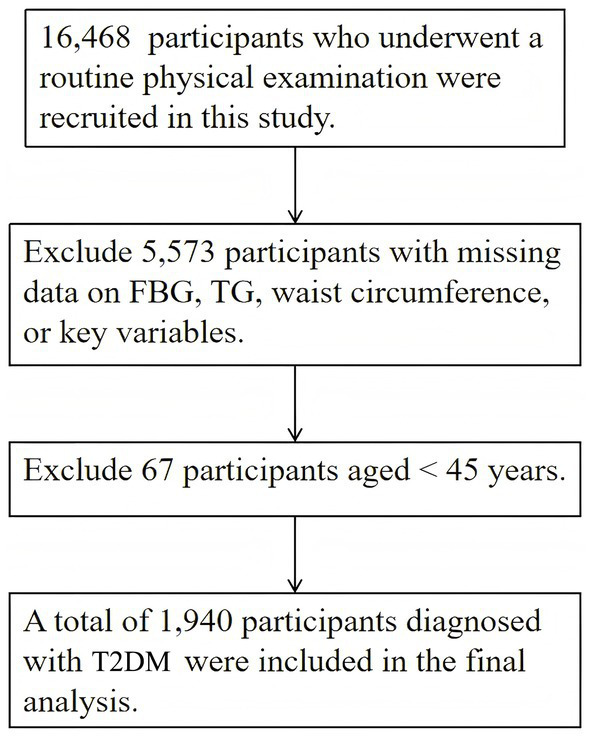
Flow of participant recruitment and exclusion in this study.

A prospective cohort design was utilized to explore the relationship between TyG-related metrics and all-cause mortality among individuals with T2DM. Data for this analysis were obtained from the Shanghai Center for Disease Control and Prevention, encompassing 16,468 participants who underwent health check-ups in 2019. A rigorous exclusion process was implemented to ensure data quality and cohort homogeneity, with detailed steps as follows: Initially, 16,468 participants were included in the preliminary screening. Participants with missing data (e.g., key variables such as fasting plasma glucose, triglycerides, or waist circumference) were excluded first, accounting for 5,573 individuals, leaving 10,895 participants. Subsequently, participants under 45 years of age (*n* = 67) were further excluded to refine the cohort, resulting in 10,828 remaining participants. Finally, 1,940 participants diagnosed with T2DM were included in the final analysis (Figure 1).

### 2.2 Definition of terms

Hypertension was defined as SBP ≥ 140 mm Hg, DBP ≥ 90 mm Hg, or the use of antihypertensive drugs. T2DM was diagnosed based on FPG levels ≥ 7. 0 mmol/L, 2 h-PG levels ≥ 11.1 mmol/L, or the use of anti-diabetic medications. Waist-to-height ratio (WHtR) was calculated by dividing waist circumference (WC) by height, while waist-to-hip ratio (WHR) was determined by dividing WC by hip circumference (HC). Definitions for the TyG index and its related composite indices are provided below. Participants were stratified into four groups (Q1, Q2, Q3, Q4) based on the quartiles of the three TyG-related indices, with Q1 designated as the reference group (19, 20).


$$\mathrm{TyG}=\ln\;[\mathrm{TG}\;(\mathrm{mg}/\mathrm{dL})\times \mathrm{FBG}\;(\mathrm{mg}/\mathrm{dL}){/}^{2}];$$

WHR=WC(cm)/HC(cm);

WHtR=WC(cm)/height(cm);

$$\mathrm{WWI}=\mathrm{WC}/\sqrt{\text{weight}}.$$

$$\text{ABSI}=\mathrm{WC}\;(m)/({\mathrm{BMI}}^{2/3}\times \text{height}\;{\left(m\right)}^{1/2});$$

TyG−WC=TyG×WC(cm);

TyG−WHR=TyG×WHR;

TyG−WHtR=TyG×WHtR;

TyG−ABSI=TyG×ABSI;

TyG−WWI=TyG×WWI.

### 2.3 Covariates

Covariates included in this study covered four domains: sociodemographic characteristics, lifestyle behaviors, laboratory biomarkers, and anthropometric indices. For sociodemographic factors, age (continuous variable, in years) and biological sex (dichotomized as male or female) were collected. Lifestyle-related covariates contained smoking status (categorized into current smokers and non-smokers), alcohol drinking status (grouped as current drinkers and non-drinkers), and regular physical activity, which was defined as engaging in structured exercise at least once per week in the month prior to baseline survey, with participants who did not meet this criterion recorded as inactive. Fasting venous blood samples were obtained following an 8–12 h overnight fast to guarantee the validity of laboratory testing. Assayed biochemical indicators included fasting blood glucose (FBG, unit: mmol/L), triglycerides (TG, unit: mmol/L), serum creatinine (Scr, unit: mmol/L), blood urea nitrogen (BUN, unit: mmol/L), total cholesterol (TC, unit: mmol/L), high-density lipoprotein cholesterol (HDL-C, unit: mmol/L), and low-density lipoprotein cholesterol (LDL-C, unit: mmol/L). Anthropometric measurements including standing height (in centimeters) and body weight (in kilograms) were collected by trained research staff according to uniform operational guidelines. These data were subsequently used to compute body mass index (BMI) and a series of TyG-derived composite indices (such as TyG-WC, TyG-WHtR) in later statistical analyses. Prior to Formal analysis, all covariates were systematically examined for missing values and extreme outliers to minimize potential bias in results.

### 2.4 Outcome measurements

All-cause mortality served as the primary endpoint of the present study. Mortality follow-up data, with a censoring date of December 31, 2024, were retrieved from the Shanghai Municipal Center for Disease Control and Prevention. Cause-specific mortality events were further classified according to the coding criteria of the International Classification of Diseases, 10th Revision (ICD-10).

### 2.5 Statistical analyses

Categorical variables were presented as counts with corresponding percentages, normally distributed continuous variables as mean (standard deviation, SD), and non-normally distributed continuous variables as median (interquartile range, IQR) for descriptive analysis of baseline characteristics. Cox proportional hazards regression models were applied to examine the associations between TyG-related indices (TyG-WC, TyG-WHR, TyG-WHtR, TyG-ABSI, TyG-WWI) and all-cause mortality among middle-aged and older adults with T2DM. Hazard ratios (HRs) and 95% confidence intervals (CIs) were calculated to quantify the magnitude of associations. Three hierarchical Cox models were established: Model 1 was an unadjusted crude model; Model 2 adjusted for sociodemographic and lifestyle factors including age, sex, smoking status, alcohol consumption, hypertension history, and regular physical activity; Model 3 additionally incorporated biochemical and anthropometric covariates on the basis of Model 2, including TC, TG, FBG, HDL-C, BUN, Scr, and body mass index (BMI). Collinearity diagnostics were performed using Pearson correlation coefficients prior to the construction of Model 3: LDL-C was excluded from further analysis due to strong collinearity with TC (correlation coefficient r > 0.7). To explore potential nonlinear associations between each TyG-related index (TyG, TyG-WC, TyG-WHR, TyG-WHtR, TyG-ABSI, TyG-WWI) and all-cause mortality, restricted cubic spline (RCS) regression was conducted with adjustments specified in Model 3. A Likelihood Ratio Test was performed to compare the fit of the linear model and the RCS model, where a two-sided *p* value < 0.05 was considered indicative of a statistically significant nonlinear trend. The predictive performance of TyG-related indices for all-cause mortality in the study population was evaluated using time-dependent receiver operating characteristic (ROC) curves. The area under the ROC curve (AUC) and its corresponding 95% CI were calculated to measure the discriminative ability of each index. Subgroup analyses stratified by sex, smoking status, alcohol consumption, and hypertension history were further performed to investigate potential effect modifications in the associations between TyG-related indices and all-cause mortality. All statistical analyses were performed using R software (version 4.5.1). A two-sided *p* value less than 0.05 was set as the threshold for statistical significance.

## 3 Results

### 3.1 Baseline characteristics of study participants according to survival status

The baseline clinical and demographic characteristics of study participants, stratified by all-cause mortality survival status during follow-up, are summarized in Table 1. A total of 1,628 survivors and 312 non-survivors were included in the analysis. Compared with survivors, non-survivors were significantly older, more likely to be male, and less physically active (all *p* < 0.001). No significant differences were observed between survivors and non-survivors in terms of current smoking status, alcohol consumption prevalence, history of hypertension, TC, TG, HDL-C, LDL-C, METS-IR, or height measurements (all *p* > 0.05). Non-survivors had significantly higher levels of FBG, BUN, and Scr, but lower weight (all *p* < 0.05). Furthermore, all central obesity-related anthropometric and metabolic indices, including ABSI, WWI, WHtR, WTR, TyG index, and the four TyG-derived composite indices evaluated in this study, were significantly elevated in non-survivors relative to survivors (all p < 0.05).

**Table 1:** Baseline characteristics of participants.

Characteristic	Survivors (*n* = 1,628)	Non-survivors (*n* = 312)	*p*-value
Age (years), median (IQR)	68.00 [63.00, 73.00]	78.00 [70.00, 84.00]	**<0.001**
Males, *n* (%)	729(44.78)	179(57.37)	**<0.001**
Current Smoking, *n* (%)	254 (15.60)	36 (11.54)	0.079
Alcohol consumption, *n* (%)	198 (12.16)	33 (10.58)	0.486
Hypertension, *n* (%)	128 (7.86)	16 (5.13)	0.116
Physical activity, *n* (%)	1,042 (64.00)	144 (46.15)	**<0.001**
FBG (mmol/L), median (IQR)	7.70 [7.00, 9.10]	8.00 [7.18, 10.10]	**0.001**
TC (mmol/L), median (IQR)	4.76 [4.26, 5.30]	4.70 [4.20, 5.23]	0.309
TG (mmol/L), median (IQR)	1.53 [1.14, 2.08]	1.55 [1.13, 2.08]	0.785
HDL-C (mmol/L), median (IQR)	1.33 [1.14, 1.56]	1.30 [1.10, 1.55]	0.181
LDL-C (mmol/L), median (IQR)	2.86 [2.47, 3.32]	2.83 [2.34, 3.35]	0.150
BUN (mmol/L), median (IQR)	5.80 [4.90, 6.80]	6.10 [5.30, 7.68]	**<0.001**
Scr (mmol/L), median (IQR)	68.00 [58.00, 80.70]	79.30 [64.00, 95.00]	**<0.001**
METS_IR [median (IQR)]	38.17 [34.63, 42.19]	38.52 [34.67, 42.71]	0.448
height, cm [median (IQR)]	162.00 [157.00, 168.00]	161.00 [156.00, 166.35]	0.120
weight, kg [median (IQR)]	65.20 [59.00, 72.00]	64.55 [58.00, 70.05]	**0.032**
ABSI [median (IQR)]	0.08 [0.08, 0.08]	0.08 [0.08, 0.09]	**<0.001**
WWI [median (IQR)]	10.66 [10.21, 11.19]	10.95 [10.42, 11.54]	**<0.001**
WHtR [median (IQR)]	0.53 [0.50, 0.57]	0.55 [0.51, 0.58]	**0.001**
WTR [median (IQR)]	0.91 [0.87, 0.95]	0.92 [0.89, 0.96]	**<0.001**
TyG [median (IQR)]	9.19 [8.84, 9.52]	9.24 [8.89, 9.63]	**0.028**
TyG_WC [median (IQR)]	794.92 [728.06, 859.41]	814.56 [750.66, 877.32]	**0.001**
TyG_WHtR [median (IQR)]	4.88 [4.49, 5.30]	5.03 [4.62, 5.48]	**<0.001**
TyG_WTR [median (IQR)]	8.35 [7.87, 8.83]	8.57 [7.95, 9.03]	**<0.001**
TyG_ABSI [median (IQR)]	0.73 [0.69, 0.77]	0.76 [0.71, 0.80]	**<0.001**
TyG_WWI [median (IQR)]	97.89 [92.49, 104.15]	100.85 [95.28, 108.39]	**<0.001**

Bold *p* values indicate significance. BUN, Blood Urea Nitrogen; Scr, Serum Creatinine; TC, Total Cholesterol; TG, Triglycerides; HDL-C, High-Density Lipoprotein Cholesterol; LDL-C, Low-Density Lipoprotein Cholesterol; FBG, Fasting Blood Glucose; BMI, Body Mass Index; IQR, Interquartile Range; WC, Waist Circumference; WHtR, Waist-to-Height Ratio; WTR, Waist-to-Thigh Ratio; ASBI, A Body Shape Index.

### 3.2 Associations of the TyG index and its composite indices with all-cause mortality

Table 2 displays the results of three hierarchical Cox proportional hazards regression models evaluating the associations of the TyG index and its composite indices with all-cause mortality in adults with T2DM. In the crude Model 1, a 1-SD increase in each of the evaluated markers was significantly associated with increased all-cause mortality risk (all HR > 1, *p* < 0.05), with TyG-ABSI demonstrating the strongest magnitude of association (HR = 1.37, 95% CI: 1.23 to 1.52). These associations remained significant in Model 2 (all HR > 1, *p* < 0.05) with slight HR reductions. In fully adjusted Model 3, neither the TyG index alone nor TyG-WTR was associated with mortality (both *p* > 0.05). In contrast, TyG-WC, TyG-WHtR, TyG-ABSI, and TyG-WWI remained independently associated with increased mortality (all HR > 1, *p* < 0.05), with TyG-WHtR being the strongest (HR = 1.44, 95%CI: 1.21–1.70). Quartile analysis showed significant dose–response trends for all indicators in Model 1 and Model 2 (all *p* for trend <0.05). In Model 3, significant trends persisted only for TyG-WC, TyG-WHtR, TyG-ABSI, and TyG-WWI (all *p* for trend<0.05), with the highest HR observed in the highest quartile of TyG-WWI (HR = 2.08, 95%CI: 1.40–3.09).

**Table 2:** Associations of the triglyceride-glucose index and its composite indices with all-cause mortality in adults with T2DM.

Exposure	HR (95% CI)		
	Model 1	Model 2	Model 3
TyG(per SD)	**1.14 (1.02,1.28)**	**1.14 (1.02,1.28)**	1.01 (0.60,1.71)
TyG-WC(per SD)	**1.21 (1.08,1.35)**	**1.13 (1.01,1.26)**	**1.24 (1.05,1.47)**
TyG-WHTR(per SD)	**1.23 (1.10,1.36)**	**1.20 (1.07,1.34)**	**1.44 (1.21,1.70)**
TyG-WTR(per SD)	**1.23 (1.10,1.38)**	**1.14 (1.02,1.28)**	1.12 (0.94,1.34)
TyG-ABSI(per SD)	**1.37 (1.23,1.52)**	**1.28 (1.15,1.43)**	**1.26 (1.11,1.43)**
TyG-WWI(per SD)	**1.35 (1.21,1.49)**	**1.31 (1.17,1.46)**	**1.32 (1.16,1.51)**
TyG (quartiles)			
Q1	1.0 (Ref)	1.0 (Ref)	1.0 (Ref)
Q2	1.08 (0.78,1.50)	1.13 (0.82,1.57)	1.08 (0.74,1.58)
Q3	1.02 (0.73,1.42)	1.04 (0.75,1.45)	1.00 (0.60,1.67)
Q4	**1.43 (1.05,1.95)**	**1.44 (1.06,1.96)**	1.32 (0.61,2.87)
*p* for trend	**0.021**	**0.020**	0.958
TyG-WC (quartiles)			
Q1	1.0 (Ref)	1.0 (Ref)	1.0 (Ref)
Q2	1.26 (0.89,1.77)	1.09 (0.77,1.55)	1.15 (0.80,1.66)
Q3	**1.64 (1.18,2.28)**	1.37 (0.98,1.91)	**1.57 (1.07,2.31)**
Q4	**1.65 (1.18,2.29)**	1.35 (0.97,1.89)	**1.64 (1.04,2.60)**
*p* for trend	**<0.001**	**0.034**	**0.012**
TyG-WHtR (quartiles)			
Q1	1.0 (Ref)	1.0 (Ref)	1.0 (Ref)
Q2	1.15 (0.82,1.62)	1.05 (0.75,1.49)	1.11 (0.77,1.60)
Q3	**1.46 (1.05,2.03)**	1.37 (0.99,1.91)	**1.66 (1.12,2.46)**
Q4	**1.64 (1.19,2.26)**	**1.53 (1.10,2.12)**	**2.06 (1.30,3.29)**
*p* for trend	**<0.001**	**0.002**	**<0.001**
TyG-WTR (quartiles)			
Q1	1.0 (Ref)	1.0 (Ref)	1.0 (Ref)
Q2	0.88 (0.62,1.24)	0.72 (0.51,1.02)	0.69 (0.48,0.99)
Q3	1.31 (0.95,1.80)	1.08 (0.78,1.49)	1.06 (0.73,1.54)
Q4	**1.51 (1.11,2.06)**	1.18 (0.86,1.61)	1.06 (0.68,1.67)
*p* for trend	**<0.001**	**0.025**	0.196
TyG-ABSI(quartiles)			
Q1	1.0 (Ref)	1.0 (Ref)	1.0 (Ref)
Q2	1.07 (0.74,1.56)	0.99 (0.68,1.44)	0.96 (0.66,1.41)
Q3	**1.94 (1.39,2.70)**	**1.68 (1.21,2.34)**	**1.68 (1.18,2.39)**
Q4	**2.03 (1.46,2.82)**	**1.72 (1.23,2.39)**	**1.57 (1.06,2.34)**
*p* for trend	**<0.001**	**<0.001**	**<0.001**
TyG-WWI (quartiles)			
Q1	1.0 (Ref)	1.0 (Ref)	1.0 (Ref)
Q2	**1.24 (0.87,1.79)**	1.14 (0.79,1.64)	1.17 (0.81,1.69)
Q3	**1.67 (1.19,2.36)**	**1.49 (1.06,2.11)**	**1.58 (1.09,2.29)**
Q4	**2.22 (1.60,3.09)**	**2.00 (1.43,2.80)**	**2.08 (1.40,3.09)**
*p* for trend	**<0.001**	**<0.001**	**<0.001**

BUN, Blood Urea Nitrogen; Scr, Serum Creatinine; TC, Total Cholesterol; TG, Triglycerides; HDL-C, High-Density Lipoprotein Cholesterol; LDL-C, Low-Density Lipoprotein Cholesterol; FBG, Fasting Blood Glucose; BMI, Body Mass Index; IQR, Interquartile Range; WC, Waist Circumference; WHtR, Waist-to-Height Ratio; WTR, Waist-to-Thigh Ratio; ASBI, A Body Shape Index. Model 1 was a crude (unadjusted) model. Model 2 was adjusted for age, sex, smoking status, alcohol consumption, hypertension history, and physical activity. Model 3 was further adjusted for blood urea nitrogen (BUN), serum creatinine (Scr), total cholesterol (TC), triglycerides (TG), high-density lipoprotein cholesterol (HDL-C), low-density lipoprotein cholesterol (LDL-C), fasting blood glucose (FBG), and body mass index (BMI), based on Model 2. Values in bold indicate statistically significant associations (*P* < 0.05).

### 3.3 Dose–response relationships between TyG-related indices and all-cause mortality

**Figure 2:**
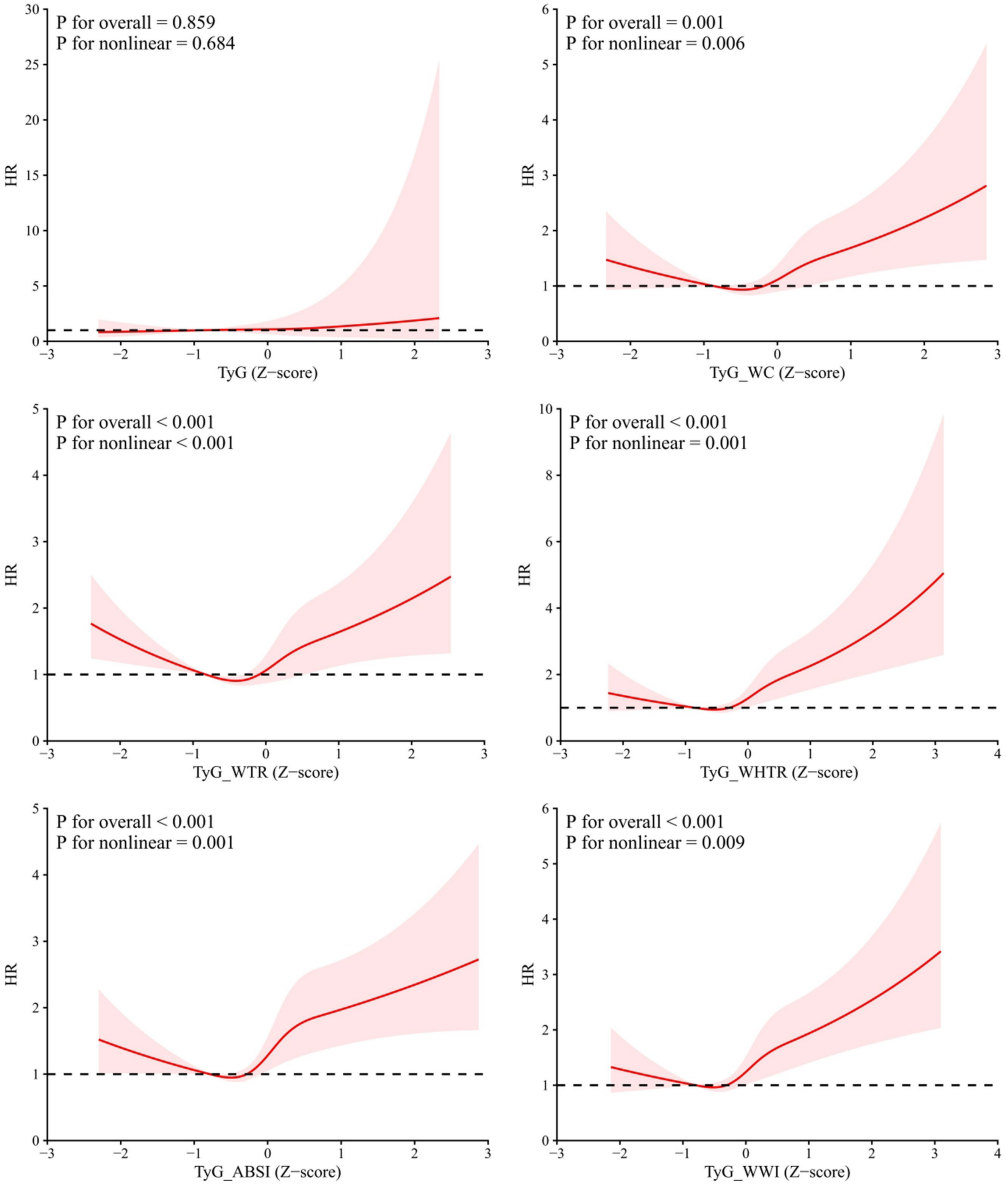
RCS analyses of the dose–response relationships between the TyG index, its composite indices, and all-cause mortality in adults with T2DM.

Restricted cubic spline analyses were performed to evaluate the dose–response relationships between the TyG index, its composite indices, and all-cause mortality under the fully adjusted Model 3 (Figure 2). The standalone TyG index showed no statistically significant overall association with mortality (*p* for overall association = 0.859), and no evidence of a nonlinear trend was observed (*p* for nonlinearity = 0.684). By contrast, all five TyG-derived composite indices (TyG-WC, TyG-WTR, TyG-WHTR, TyG-ABSI, TyG-WWI) demonstrated highly significant overall associations with mortality (all *p* for overall < 0.001), and all showed statistically significant nonlinear dose–response relationships (all *p* for nonlinearity < 0.01). The spline curves presented a consistent J-shaped trajectory across all composite indices: hazard ratios reached their minimum at Z-scores close to 0, and mortality risk increased rapidly with higher index values, especially for TyG-WHTR, TyG-ABSI and TyG-WWI.

### 3.4 Predictive performance of the TyG index and its composite indices for all-cause mortality

Time-dependent area under the receiver operating characteristic curve (AUC) values for the TyG index and its composite indices in predicting 1-year, 3-year and 5-year all-cause mortality among patients with T2DM are presented in Supplementary Table S1. All indices exhibited moderate discriminative ability across the three time points, with AUC values ranging from 0.70 to 0.74. At 1-year follow-up, TyG-ABSI and TyG-WWI achieved the highest AUC (0.74), whereas the standalone TyG index yielded a slightly lower AUC (0.72). At both 3- and 5-year follow-up, the predictive performance remained relatively stable, and TyG-ABSI and TyG-WWI continued to display numerically higher AUC values than the other indices. Collectively, composite indices integrating TyG with anthropometric parameters, particularly TyG-ABSI and TyG-WWI, showed slightly superior predictive capacity compared with the TyG index alone at all time points, supporting their potential utility in long-term mortality risk stratification among adults with T2DM.

### 3.5 Subgroup analyses of TyG-related indices and all-cause mortality

In the age-stratified subgroup analysis, all evaluated TyG-related indices (TyG, TyG-WC, TyG-WHtR, TyG-WTR, TyG-ABSI, TyG-WWI) were significantly associated with higher all-cause mortality in participants aged >65 years (all *p* < 0.05), while no significant associations were found in those ≤65 years (all *p* > 0.05). Among non-drinkers, non-smokers, and participants without a history of hypertension, most TyG composite indices remained significantly associated with a higher mortality risk. In both male and female subgroups, TyG-ABSI and TyG-WWI exhibited consistent and significant associations with all-cause mortality. By contrast, TyG-WTR was significantly associated with all-cause mortality only in current smokers (Supplementary Tables S2–S7). Collectively, these subgroup analyses confirmed the robustness and consistency of TyG composite indices—particularly TyG-ABSI, TyG-WHtR, and TyG-WWI—in predicting all-cause mortality among adults with T2DM.

## 4 Discussion

### 4.1 Key findings

In this diabetic cohort, non-survivors with T2DM exhibited higher levels of metabolic dysfunction and central obesity-related indices. The standalone TyG index showed no independent association with all-cause mortality after full adjustment, whereas TyG-WC, TyG-WHtR, TyG-ABSI, and TyG-WWI remained significantly predictive. These results align with prior reports that combined metabolic-anthropometric indices improve risk prediction (15, 21, 22). The J-shaped dose–response relationships further support the nonlinear association between TyG composite indices and mortality.

Collectively, our study identified a pronounced J-shaped dose–response association between all TyG-related composite indices and all-cause mortality risk in the diabetic cohort. Of note, the linearity and curvature of such relationships often differ across outcomes and study populations, leading to inconsistent findings among published investigations. Consistent with this heterogeneity in dose–response patterns, existing literature has documented varied curve shapes for the link between TyG-related markers and adverse outcomes: for example, several previous investigations identified U-shaped or L-shaped associations between TyG-derived indices and all-cause or cardiovascular mortality across populations with prediabetes, diabetes, hypertension, or metabolic dysfunction-associated steatotic liver disease (12, 23, 24). In contrast to these nonlinear findings, work by Dang and colleagues in a U. S. cohort observed largely linear relationships between all four TyG-related composite indices and mortality risk (25). In cohorts of patients diagnosed with non-alcoholic fatty liver disease, the associations of TyG and TyG-WHtR with cardiovascular event risk mostly follow a linear trend, while a nonlinear relationship was identified for all-cause mortality in the same population (13). This variability in dose–response patterns across different outcomes and populations can be partially attributed to the multifaceted pathophysiological features of metabolic syndrome, which include dysregulated glucose and lipid metabolism, insulin resistance, persistent low-grade inflammatory state, and aberrant adipose tissue distribution (26, 27).

### 4.2 Potential biological mechanisms

The mechanisms underlying the observed associations involve the integrated dysfunction of the broader cardiometabolic network, not limited to isolated insulin resistance. The TyG index captures insulin resistance, lipid metabolism disorders, and correlates with low-grade inflammation and innate immune activation. When combined with anthropometric indicators reflecting central obesity, adipose tissue dysfunction and inflammatory burden, the composite indices jointly drive endothelial injury, elevated oxidative stress and subclinical atherosclerosis, increasing long-term mortality risk in people with diabetes. Notably, both extremely low and excessively high TyG-related composite index levels signal unstable metabolic states: abnormally low levels reflect impaired compensatory metabolic regulation, while excessively high levels indicate exacerbated metabolic dysregulation coupled with abnormal myeloid inflammatory activation, both linked to higher cardiovascular and mortality risk (28). Central obesity and visceral fat accumulation further amplify these adverse effects via reciprocal interactions between pro-inflammatory cytokine secretion, innate immune pathway activation and endothelial dysfunction (29, 30), with myeloid activation-related inflammatory markers confirmed to correlate with early vascular damage (31). These findings highlight the interplay of metabolic, adipose and inflammatory-immune pathways in driving the observed associations, which can inform improved risk stratification for diabetic populations.

### 4.3 Clinical implications

For adults with T2DM, early stratification of all-cause mortality risk is a core clinical priority, as concurrent metabolic dysregulation, visceral adiposity, adipose tissue dysfunction and chronic inflammatory burden often act synergistically to drive vascular injury and worsen long-term survival outcomes. Multiple simple surrogate markers of insulin resistance have been developed for risk stratification, including the well-validated TG/HDL ratio, which was recently linked to arterial stiffness in prediabetic populations (32) and primarily reflects lipid metabolism imbalance. Recent evidence also confirms that direct vascular damage markers, including pulse wave velocity (PWV), intima-media thickness (IMT) and carotid atherosclerosis, are strongly associated with cardiovascular risk stratified by SCORE2-Diabetes, highlighting the important value of vascular damage assessment in refining risk stratification for T2DM patients (33).

Our cohort study finds that after full adjustment, TyG-derived composite indices retain significant predictive value for mortality, while the standalone TyG index does not. This superior performance stems from the ability of these composite indices, which combine the TyG parameter (integrating both glucose and lipid metabolism abnormalities to reflect metabolic dysregulation and low-grade inflammatory activation) with anthropometric measures (WC, WHtR, ABSI, WWI, reflecting visceral adiposity and adipose dysfunction), to integrate the combined burden of metabolic and inflammatory alterations. As supported by prior evidence, such integrated cardiometabolic markers more accurately reflect overall disease burden and correlate more closely with subclinical vascular damage than single metabolic markers (33). These indices can serve as complementary, easy-to-use tools within existing clinical risk stratification frameworks (including SCORE2-Diabetes), helping clinicians identify high-risk patients earlier, especially in settings without access to routine vascular damage assessment, and inform targeted interventions targeting both metabolic and inflammatory pathways to improve long-term outcomes.

These integrated markers can be seamlessly integrated into routine clinical workflows for T2DM management, as fasting blood glucose (FBG), triglyceride (TG) levels, and the required anthropometric parameters (including WC and WHtR) are already routinely collected as part of standard care for diabetic populations. TyG-related indices can be calculated automatically within hospital electronic health record systems, enabling real-time identification of patients at high mortality risk, who can then undergo further targeted vascular damage assessments for more precise management. Against the global trend of population aging, these simple, low-cost, and highly scalable indicators carry important public health value for large-scale mortality risk screening among diabetic adults, especially in community and primary care settings where access to advanced diagnostic resources is often limited. Integrating TyG-WC, TyG-WHtR, TyG-ABSI, and TyG-WWI into existing T2DM and cardiovascular risk assessment tools can complement traditional metabolic and obesity-related evaluations, inform the development of population-level mortality prevention policies, and ultimately help reduce the growing mortality burden associated with diabetes in aging, high-prevalence populations. Future research will further compare the prognostic performance of TG/HDL-based composite indices with the TyG-derived markers evaluated in this study, and explore combined models integrating simple metabolic indices and vascular damage markers to optimize T2DM risk stratification systems.

### 4.4 Strengths and limitations

Our investigation has several notable methodological strengths. First, we adopted an innovative approach to combine the TyG index—a well-validated surrogate marker of insulin resistance—with multiple core anthropometric parameters to develop composite risk indices, which enable a more holistic assessment of mortality risk compared to using either single metabolic or anthropometric markers alone. Second, our analysis focused specifically on all-cause mortality, a clinically meaningful hard endpoint in the adult diabetic population, which ensures the study findings have direct translational value for routine clinical risk stratification practice. Third, we applied rigorous statistical adjustments for a wide range of potential confounding variables, and conducted multiple sensitivity analyses to confirm the robustness and stability of the observed associations. Nevertheless, several limitations should be acknowledged when interpreting our results. First, the observational study design prevents us from establishing causal inferences between TyG-derived composite indices and all-cause mortality in diabetic adults, and residual confounding from unmeasured or unaccounted factors cannot be fully excluded. Second, we did not perform subgroup analyses stratified by diabetes subtype (type 1 vs. type 2) or disease duration, and only baseline TyG-related index values were collected for analysis, with no repeated measurements during follow-up to capture dynamic changes in these markers over time. Third, potential selection bias may have been introduced due to the single-center study setting, and we did not conduct head-to-head comparisons of the predictive performance of our composite indices with existing, well-established mortality risk prediction models. Further research is warranted to address these gaps. Future studies with prospective designs incorporating repeated measurements of TyG-related indices during follow-up will help clarify the causal nature of the observed associations. Additional studies including stratified subgroup analyses, direct performance comparisons with established risk prediction tools, interventional studies evaluating the impact of index-guided management strategies, and multi-center cohorts with diverse demographic and clinical characteristics are needed to enhance the generalizability of our findings and further validate the clinical utility of TyG-based composite indices in real-world practice.

## 5 Conclusion

In adults with T2DM, four TyG-anthropometric composite indices (TyG-WC, TyG-WHtR, TyG-ABSI, TyG-WWI) are independently associated with higher all-cause mortality risk (with J-shaped dose–response relationships), while the standalone TyG index shows no significant association. These stable, easy-to-use composite indices, which capture integrated cardiometabolic burden, can serve as complementary tools for existing risk stratification to support early identification of high-risk patients.

## Data Availability

The original contributions presented in the study are included in the article/Supplementary material, further inquiries can be directed to the corresponding author/s.
