# Supplementary material for: Associations of the triglyceride-glucose index and its composite indices with mortality in adults with T2DM
Source: Front Nutr. 2026 May 11;13:1822295. doi: 10.3389/fnut.2026.1822295 (PMC13199349; doi:10.3389/fnut.2026.1822295)
Supplement: Supplementary file 1 [file Supplementary_file_1.docx]

TableS1. Time-dependent AUC values of the TyG index and its composite indices for predicting all-cause mortality

| **variable** | **time_days** | **AUC** | **Lower** | **Upper** |
| --- | --- | --- | --- | --- |
| TyG | 365 | 0.72 | 0.63 | 0.81 |
| TyG | 1095 | 0.70 | 0.66 | 0.74 |
| TyG | 1825 | 0.71 | 0.68 | 0.74 |
| TyG-WC | 365 | 0.73 | 0.64 | 0.83 |
| TyG-WC | 1095 | 0.70 | 0.66 | 0.74 |
| TyG-WC | 1825 | 0.71 | 0.67 | 0.74 |
| TyG-WTR | 365 | 0.73 | 0.64 | 0.82 |
| TyG-WTR | 1095 | 0.70 | 0.66 | 0.75 |
| TyG-WTR | 1825 | 0.71 | 0.68 | 0.74 |
| TyG-WHTR | 365 | 0.73 | 0.63 | 0.82 |
| TyG-WHTR | 1095 | 0.70 | 0.66 | 0.75 |
| TyG-WHTR | 1825 | 0.71 | 0.68 | 0.74 |
| TyG-ABSI | 365 | 0.74 | 0.64 | 0.83 |
| TyG-ABSI | 1095 | 0.71 | 0.67 | 0.75 |
| TyG-ABSI | 1825 | 0.72 | 0.68 | 0.75 |
| TyG-WWI | 365 | 0.73 | 0.64 | 0.83 |
| TyG-WWI | 1095 | 0.71 | 0.67 | 0.75 |
| TyG-WWI | 1825 | 0.72 | 0.68 | 0.75 |

Abbreviations: AUC, area under the receiver operating characteristic curve; TyG, triglyceride-glucose index; TyG-WC, triglyceride-glucose index combined with waist circumference; TyG-WTR, triglyceride-glucose index combined with waist-to-thigh ratio; TyG-WHtR, triglyceride-glucose index combined with waist-to-height ratio; TyG-ABSI, triglyceride-glucose index combined with a body shape index; TyG-WWI, triglyceride-glucose index combined with waist-to-hip ratio.

Table S2. Subgroup analyses of the associations between TyG and all-cause mortality in adults with T2DM (Model 3)

| **Variables** | **Subgroup** | **Level** | **HR Per SD** | **P for Interaction** |
| --- | --- | --- | --- | --- |
| TyG | Age Group | ≤65 | 1.15(0.81,1.65) | 0.671 |
| TyG | Age Group | ＞65 | **1.13(1.00,1.27)** |  |
| TyG | Alcohol Consumption | no | **1.13(1.01,1.28)** | 0.693 |
| TyG | Alcohol Consumption | yes | 1.13(0.81,1.58) |  |
| TyG | Gender | male | 1.09(0.94,1.26) | 0.295 |
| TyG | Gender | female | **1.23(1.03,1.47)** |  |
| TyG | Hypertension | no | **1.14(1.02,1.28)** | 0.900 |
| TyG | Hypertension | yes | 0.89(0.56,1.42) |  |
| TyG | Current Smoking | no | **1.13(1.00,1.27)** | 0.564 |
| TyG | Current Smoking | yes | 1.26(0.91,1.73) |  |

Abbreviations: TyG, triglyceride-glucose index; HR, hazard ratio; SD, standard deviation.

Values in bold indicate statistically significant associations (*P* < 0.05).

Table S3. Subgroup analyses of the associations between TyG-WC and all-cause mortality in adults with T2DM (Model 3)

| **Variables** | **Subgroup** | **Level** | **HR Per SD** | **P for Interaction** |
| --- | --- | --- | --- | --- |
| TyG-WC | Age Group | ≤65 | 1.15(0.78,1.69) | 0.696 |
| TyG-WC | Age Group | ＞65 | **1.12(1.00,1.26)** |  |
| TyG-WC | Alcohol Consumption | no | **1.16(1.03,1.30)** | 0.153 |
| TyG-WC | Alcohol Consumption | yes | 0.89(0.62,1.27) |  |
| TyG-WC | Gender | male | **1.17(1.00,1.37)** | 0.546 |
| TyG-WC | Gender | female | 1.09(0.93,1.27) |  |
| TyG-WC | Hypertension | no | **1.13(1.01,1.27)** | 0.822 |
| TyG-WC | Hypertension | yes | 0.96(0.55,1.70) |  |
| TyG-WC | Current Smoking | no | **1.14(1.02,1.29)** | 0.46 |
| TyG-WC | Current Smoking | yes | 1.01(0.72,1.42) |  |

Abbreviations: TyG-WC, triglyceride-glucose index combined with waist circumference; HR, hazard ratio; SD, standard deviation.

Values in bold indicate statistically significant associations (*P* < 0.05).

Table S4. Subgroup analyses of the associations between TyG-WHTR and all-cause mortality in adults with T2DM (Model 3)

| **Variables** | **Subgroup** | **Level** | **HR Per SD** | **P for Interaction** |
| --- | --- | --- | --- | --- |
| TyG-WHTR | Age Group | ≤65 | 1.24(0.84,1.84) | 0.825 |
| TyG-WHTR | Age Group | ＞65 | **1.19(1.06,1.34)** |  |
| TyG-WHTR | Alcohol Consumption | no | **1.22(1.08,1.37)** | 0.271 |
| TyG-WHTR | Alcohol Consumption | yes | 0.98(0.68,1.41) |  |
| TyG-WHTR | Gender | male | **1.28(1.09,1.50)** | 0.32 |
| TyG-WHTR | Gender | female | 1.13(0.97,1.32) |  |
| TyG-WHTR | Hypertension | no | **1.20(1.07,1.35)** | 0.911 |
| TyG-WHTR | Hypertension | yes | 1.00(0.56,1.79) |  |
| TyG-WHTR | Current Smoking | no | **1.20(1.07,1.35)** | 0.698 |
| TyG-WHTR | Current Smoking | yes | 1.16(0.83,1.62) |  |

Abbreviations: TyG-WHtR, triglyceride-glucose index combined with waist-to-height ratio; HR, hazard ratio; SD, standard deviation.

Values in bold indicate statistically significant associations (*P*< 0.05).

Table S5. Subgroup analyses of the associations between TyG-WTR and all-cause mortality in adults with T2DM (Model 3)

| **Variables** | **Subgroup** | **Level** | **HR Per SD** | **P for Interaction** |
| --- | --- | --- | --- | --- |
| TyG-WTR | Age Group | ≤65 | 1.23(0.85,1.77) | 0.474 |
| TyG-WTR | Age Group | ＞65 | **1.13(1.00,1.27)** |  |
| TyG-WTR | Alcohol Consumption | no | 1.12(0.99,1.26) | 0.345 |
| TyG-WTR | Alcohol Consumption | yes | 1.27(0.90,1.79) |  |
| TyG-WTR | Gender | male | 1.16(0.98,1.36) | 0.822 |
| TyG-WTR | Gender | female | 1.13(0.95,1.33) |  |
| TyG-WTR | Hypertension | no | **1.15(1.02,1.29)** | 0.885 |
| TyG-WTR | Hypertension | yes | 0.87(0.52,1.47) |  |
| TyG-WTR | Current Smoking | no | 1.10(0.97,1.24) | 0.089 |
| TyG-WTR | Current Smoking | yes | **1.50(1.06,2.11)** |  |

Abbreviations: TyG-WTR, triglyceride-glucose index combined with waist-to-thigh ratio; HR, hazard ratio; SD, standard deviation.

Values in bold indicate statistically significant associations (*P* < 0.05).

Table S6. Subgroup analyses of the associations between TyG-ABSI and all-cause mortality in adults with T2DM (Model 3)

| **Variables** | **Subgroup** | **Level** | **HR Per SD** | **P for Interaction** |
| --- | --- | --- | --- | --- |
| TyG-ABSI | Age Group | ≤65 | 1.32(0.89,1.97) | 0.583 |
| TyG-ABSI | Age Group | ＞65 | **1.27(1.14,1.43)** |  |
| TyG-ABSI | Alcohol Consumption | no | **1.30(1.16,1.45)** | 0.465 |
| TyG-ABSI | Alcohol Consumption | yes | 1.18(0.86,1.61) |  |
| TyG-ABSI | Gender | male | **1.30(1.12,1.51)** | 0.852 |
| TyG-ABSI | Gender | female | **1.27(1.09,1.47)** |  |
| TyG-ABSI | Hypertension | no | **1.30(1.17,1.45)** | 0.277 |
| TyG-ABSI | Hypertension | yes | 0.72(0.37,1.40) |  |
| TyG-ABSI | Current Smoking | no | **1.28(1.14,1.43)** | 0.977 |
| TyG-ABSI | Current Smoking | yes | 1.28(0.95,1.73) |  |

Abbreviations: TyG-ABSI, triglyceride-glucose index combined with a body shape index; HR, hazard ratio; SD, standard deviation.

Values in bold indicate statistically significant associations (*P* < 0.05).

Table S7. Subgroup analyses of the associations between TyG-WWI and all-cause mortality in adults with T2DM (Model 3)

| Variables | Subgroup | Level | HR Per SD | P for Interaction |
| --- | --- | --- | --- | --- |
| TyG-WWI | Age Group | ≤65 | 1.38(0.91,2.08) | 0.662 |
| TyG-WWI | Age Group | ＞65 | **1.30(1.16,1.45)** |  |
| TyG-WWI | Alcohol Consumption | no | **1.32(1.18,1.49)** | 0.402 |
| TyG-WWI | Alcohol Consumption | yes | 1.16(0.84,1.59) |  |
| TyG-WWI | Gender | male | **1.36(1.17,1.59)** | 0.505 |
| TyG-WWI | Gender | female | **1.25(1.08,1.46)** |  |
| TyG-WWI | Hypertension | no | **1.32(1.18,1.47)** | 0.486 |
| TyG-WWI | Hypertension | yes | 0.82(0.43,1.56) |  |
| TyG-WWI | Current Smoking | no | **1.30(1.16,1.46)** | 0.922 |
| TyG-WWI | Current Smoking | yes | 1.31(0.97,1.77) |  |

Abbreviations:TyG-WWI, triglyceride-glucose index combined with waist-to-hip ratio. HR, hazard ratio; SD, standard deviation.

Values in bold indicate statistically significant associations (*P*< 0.05).
